# Nonlinear joint models for individual dynamic prediction of risk of death using Hamiltonian Monte Carlo: application to metastatic prostate cancer

**DOI:** 10.1186/s12874-017-0382-9

**Published:** 2017-07-17

**Authors:** Solène Desmée, France Mentré, Christine Veyrat-Follet, Bernard Sébastien, Jérémie Guedj

**Affiliations:** 1 IAME, UMR 1137, INSERM, Université Paris Diderot, Sorbonne Paris Cité, 16, rue Henri Huchard, Paris, 75018 France; 2 Translational Informatics, Translational Medicine, Sanofi, Bridgewater, USA; 3 Clinical Trial Simulation, Sanofi, Chilly-Mazarin, France

**Keywords:** Calibration, Discrimination, Hamiltonian Monte Carlo, Individual dynamic prediction, Nonlinear joint model

## Abstract

**Background:**

Joint models of longitudinal and time-to-event data are increasingly used to perform individual dynamic prediction of a risk of event. However the difficulty to perform inference in nonlinear models and to calculate the distribution of individual parameters has long limited this approach to linear mixed-effect models for the longitudinal part. Here we use a Bayesian algorithm and a nonlinear joint model to calculate individual dynamic predictions. We apply this approach to predict the risk of death in metastatic castration-resistant prostate cancer (mCRPC) patients with frequent Prostate-Specific Antigen (PSA) measurements.

**Methods:**

A joint model is built using a large population of 400 mCRPC patients where PSA kinetics is described by a biexponential function and the hazard function is a PSA-dependent function. Using Hamiltonian Monte Carlo algorithm implemented in Stan software and the estimated population parameters in this population as priors, the *a posteriori* distribution of the hazard function is computed for a new patient knowing his PSA measurements until a given landmark time. Time-dependent area under the ROC curve (AUC) and Brier score are derived to assess discrimination and calibration of the model predictions, first on 200 simulated patients and then on 196 real patients that are not included to build the model.

**Results:**

Satisfying coverage probabilities of Monte Carlo prediction intervals are obtained for longitudinal and hazard functions. Individual dynamic predictions provide good predictive performances for landmark times larger than 12 months and horizon time of up to 18 months for both simulated and real data.

**Conclusions:**

As nonlinear joint models can characterize the kinetics of biomarkers and their link with a time-to-event, this approach could be useful to improve patient’s follow-up and the early detection of most at risk patients.

**Electronic supplementary material:**

The online version of this article (doi:10.1186/s12874-017-0382-9) contains supplementary material, which is available to authorized users.

## Background

One potential major application of mathematical modelling to personalized medicine is to provide dynamic prediction of a disease progression and occurrence of a severe clinical event. For that purpose an increasingly popular approach in the statistical community is to use joint models, which simultaneously handle longitudinal and time-to-event data by maximizing the joint likelihood of both processes to avoid the bias due to informative dropouts or measurements error [1–3]. In this approach, the longitudinal part is described by a mixed-effect model and the survival part is described by a parametric or semi-parametric hazard function which depends on the true, unobserved, biomarker kinetics. After the parameters have been estimated, they can be used as priors to make “individual dynamic predictions” of the disease progression [4–6], i.e., prospective predictions that can be updated over the patient’s follow-up. Nevertheless the numerical difficulties have long limited the use of joint models, and hence of dynamic predictions, to linear models for the longitudinal processes. Although this approach can be made more flexible by using splines [3, 7], it does not handle models that are nonlinear in the parameters, i.e., nonlinear mixed-effect models (NLMEM), such as mechanistic models defined by differential equations.

Recently, we and others have shown that the SAEM algorithm, initially developed for inference in NLMEM, could be used to provide precise estimates of nonlinear joint models [8, 9]. However, even after the population parameters have been estimated, one still needs to characterize the entire *a posteriori* distribution of the individual parameters, which has no analytical form, in order to perform individual dynamic predictions. In that context, Bayesian inference using Markov Chain Monte Carlo (MCMC) appears naturally suited. Nevertheless, traditional MCMC are based on random walk which provides estimators with good properties of convergence, but in practice and especially in a high-dimensional context, this asymptotic behavior is of limited use because of finite computational resources. In that case, the Halmitonian Monte Carlo (HMC), implemented in Stan [10], uses the geometry of the parameters space to generate effective and rapid exploration of this space, and stronger guarantees on the convergence [11–14].

Here we propose to use nonlinear joint model and HMC to characterize the *a posteriori* distribution of the individual survival probability. We apply in this nonlinear context novel tools that have been developed to assess time-dependent discrimination and calibration metrics of dynamic models, such as the Area under the Receiver Operating Characteristic (ROC) curve (AUC) and the Brier score (BS) [5, 15, 16].

The approach is applied to a phase III clinical trial in metastatic castration-resistant prostate cancer (mCRPC) patients [17] where prostate-specific antigen (PSA) is frequently measured. The priors are obtained using a nonlinear joint model whose parameters are estimated on a training dataset of 400 patients using the SAEM algorithm implemented in Monolix. We show how dynamic predictions can be performed by characterizing the full *a posteriori* distribution of the risk of death for new individuals using 200 simulated patients and 196 mCRPC patients from a validation dataset. The time-dependent metrics for discrimination and calibration are obtained for different landmark and horizon times and we discuss the potential applications of this approach.

## Methods

### General framework

#### Joint model with a nonlinear longitudinal biomarker

Let *N* the number of patients and $y_{i}=\{y_{i1},\ {\ldots },\ y_{in_{i}}\}\phantom {\dot {i}\!}$ the vector of the longitudinal observations in patient *i*, where *y*_*ij*_ denotes the *j* ^*th*^ measurement of the biomarker for the individual *i* at time *t*_*ij*_, where *i*=1,…, *N*, *j*=1,…, *n*_*i*_, and *n*_*i*_ is the total number of measurements in subject *i*. The observations are given by:

1 $$  y_{ij}={b}(t_{ij},\ \psi_{i})+e_{ij}  $$

where *b*(*t*, *ψ*_*i*_) is the true and unobserved value of the biomarker at time point *t*, nonlinear in regards to the individual parameters *ψ*_*i*_, and *e*_*ij*_ is the residual Gaussian error of mean 0 and variance *σ* ^2^. *ψ*_*i*_=*g*(*μ*,*η*_*i*_) depends on the fixed effects *μ* identical for all patients and on the random effects *η*_*i*_ specific for each individual, and the function *g* defines the transformations of the individual parameters. The random effects are assumed to be normally distributed with mean zero and variance-covariance matrix *Ω* ($\eta _{i}\sim \mathcal {N}(0,\ \Omega)$), and are assumed independent of the residual error *e*_*ij*_.

Let *X*_*i*_ denote the time-to-event and *C*_*i*_ the censoring time for the patient *i*. Only *T*_*i*_=min(*X*_*i*_, *C*_*i*_) is observed and we note *δ*_i_=1 if X_i_≤C_i_ and 0 otherwise. The individual hazard function of the risk of death can be written as follows:

$$h_{i}\left(t|{\mathbf{B}}\left(t,\ \psi_{i}\right)\right)=h_{0}(t){\exp \left(\beta\ f\!\!\left(t,\ \psi_{i}\right)+\gamma^{T}w_{i}\right)\ } $$

where **B**(*t*, *ψ*_*i*_)={*b*(*u*, *ψ*_*i*_);0≤*u*<*t*} denotes the history of the true unobserved longitudinal process up to t, *h*_0_ is a parametric baseline hazard function of vector of parameters denoted *p*_0_, and *γ* is the vector of coefficients associated with a vector of baseline covariates *w*_*i*_. The link function *f* depends on the true biomarker kinetics **B**(*t*, *ψ*_*i*_) and the parameter *β* quantifies the strength of the association between the biomarker kinetics and the risk of event.

The log-likelihood for subject *i* is given by:

2 $$  LL_{i}(\theta)=\log\int p(y_{i}|\eta_{i};\theta)p(T_{i},\delta_{i}|\eta_{i};\theta)p(\eta_{i};\theta)d\eta_{i}  $$

where *θ*={*μ*,*Ω*,*σ*,*p*_0_,*γ*,*β*} is the vector of parameters to estimate, *p*(*y*_*i*_|*η*_*i*_;*θ*) is the probability density function of the longitudinal observations conditionally on the random effects *η*_*i*_, *p*(*η*_*i*_;*θ*) is the probability density function of the random effects and

$$p(T_{i},\delta_{i}|\eta_{i};\theta)=h_{i}(T_{i}|\mathbf{B}(T_{i},\psi_{i});\theta)^{\delta_{i}}S_{i}(T_{i}|\psi_{i};\theta) $$

is the likelihood of the survival part, with:

3 $$ \begin{aligned} S_{i}\left(t\mathrel{\left|\vphantom{t \psi_{i} ;\theta}\right.\kern-\nulldelimiterspace}\psi_{i} ;\theta\right)&=\mathbf{P}(X_{i}>t|\mathbf{B}\left(t,\ \psi_{i}\right) ; \theta)\\&=\text{exp}\left(-\int^{t}_{0}{h_{i}\left(u|{\mathbf B}\left(t,\ \psi_{i}\right) ;\theta\right)}du\right) \end{aligned}  $$

the survival function conditionally on the random effects.

In a NLMEM framework, the Stochastic Approximation Expectation-Maximization (SAEM) algorithm [18] implemented in Monolix (www.lixoft.eu) provides unbiased estimates for both longitudinal and survival parameters [8, 9]. As in other EM algorithms, this algorithm is an iterative process where each iteration is divided into a step where the complete likelihood conditional on observations is calculated (E-step), and a step where the complete likelihood is maximized (M-step). In addition, in the SAEM algorithm, the E-step is divided into two parts: a simulation of individual parameters using a Markov Chain Monte Carlo (MCMC) algorithm (S-step), and then a calculation of the expected likelihood using a stochastic approximation (A-step). Once parameters are estimated, the Fisher information matrix (FIM) can be stochastically approximated to obtain the relative standard errors (r.s.e).

#### Individual dynamic predictions

Let assume that the set of parameters *θ* has been previously estimated in a large dataset, called “training dataset” hereafter. Now we are interested in a new subject *i* with longitudinal biomarker measurements available until a landmark time *s*≥0: $\mathcal {Y}_{i}(s)=\{y_{ij} ;\ 0\leq t_{ij}\leq s\}$, for whom we aim to predict the risk of death until time *s*+*t*, where *t*>0 is called the horizon time. Since the patient is alive at time *s*, we focus on the conditional probability of death between *s* and the prediction horizon *s*+*t* given by:

$$\pi_{i}\left(s+t\mathrel{\left| \right.\kern-\nulldelimiterspace}s\right)= \mathbf{P}(X_{i}<s+t|X_{i}>s,~\mathcal{Y}_{i}(s) ;\ \theta) $$

For each landmark time *s*, the biomarker measurements of patient *i* up to time *s* are used to compute the *a posteriori* distribution of the individual parameters and infer the survival probability with a prediction interval taking into account the uncertainty of the individual parameter estimation. For that purpose, a Monte Carlo estimate of *π*_*i*_(*s*+*t*|*s*) can be obtained using the Bayesian approach proposed by Rizopoulos (2011) [4] which consists in repeating *L* times the following loop, where *L* denotes the number of Monte Carlo realizations: for *ℓ*=1,…,*L*,

1. Draw a realization $\eta ^{(\ell)}_{i}$ from the posterior distribution of the random effects:
  4 $${} {{\begin{aligned} \left\{\eta_{i} | \ X_{i}>s,\mathcal{Y}_{i}(s) ;\ \theta\right\} \sim \left\{\!\prod\limits_{j=1}^{n_{i}(s)} p(y_{ij}\ |\ \eta_{i}; \theta)\!\right\}\! S_{i}\!\left(s\ | \ g(\mu, \eta_{i}); \theta\right)p(\eta_{i}; \theta) \end{aligned}}}  $$
  where *n*_*i*_(*s*) is the number of longitudinal measurements of patient *i* available at the landmark time *s*.
2. Infer $\psi ^{(\ell)}_{i} = g\left (\mu, \eta ^{(\ell)}_{i} \right)$
3. Compute: $y^{(\ell)}_{i}(u)=b\left (u,\psi ^{(\ell)}_{i}\right)$ with *u*>0
  And: $\pi ^{(\ell)}_{i}\left (s+t\mathrel {\left |\right.\kern -\nulldelimiterspace }s\right)=\frac {S_{i}\left (s\mathrel {\left |\right.\kern -\nulldelimiterspace }\psi ^{(\ell)}_{i} ; \theta \right)-S_{i}\left (s+t\mathrel {\left |\right.\kern -\nulldelimiterspace }\psi ^{(\ell)}_{i} ; \theta \right)}{S_{i}\left (s\mathrel {\left |\right.\kern -\nulldelimiterspace }\psi ^{(\ell)}_{i} ;\theta \right)}$

The difficulty is the first step since the posterior distribution of the random effects has no analytical solution when the model for the biomarker is nonlinear in regard to the parameters. We use Hamiltonian Monte Carlo (HMC) algorithm implemented in Stan software version 2.8 [10] and its R (version 3.1.3) interface. The *a priori* distribution of the random effects is assumed to be normal of mean zero and variance-covariance matrix *Ω*, estimated on the training dataset. The *a posteriori* distribution of the individual random effects defined by the Eq. (4) requires the integration of the hazard function in Eq. (3) of the survival. For that purpose, we use a Gauss-Legendre quadrature of order 8. Of note, and unlike what was proposed by Rizopoulos (2011) [4] the uncertainty in *θ* is neglected (see “Results”).

The *L* realizations $\left \{y^{(\ell)}_{i}(u), \ell =1, \dots, L\right \}$ and $\left \{\pi ^{(\ell)}_{i}\left (s+t\mathrel {\left |\right.\kern -\nulldelimiterspace }s\right), \ell =1, \dots, L\right \}$ can be used to derive estimates of the biomarker kinetics and estimates of *π*_*i*_(*s*+*t*|*s*) as:

$${\hat{y}}_{i}(u)=median\left\{y^{(\ell)}_{i}(u),\ \ell=1,\ \dots,\ L\right\} $$

And:

5 $$ {\begin{aligned} {\hat{\pi}}_{i}\left(s+t\mathrel{\left|\right.\kern-\nulldelimiterspace}s\right)=median\left\{\pi^{(\ell)}_{i}\left(s+t\mathrel{\left|\right.\kern-\nulldelimiterspace}s\right), \ell=1,\ \dots,\ L {\vphantom{\pi^{(\ell)}_{i}}}\right\} \end{aligned}}  $$

And 95% prediction intervals for the individual predictions are obtained using the 2.5% and 97.5% Monte Carlo sample percentiles.

#### Model discrimination and calibration

The discrimination, i.e., the capacity of the model to distinguish patients of low and high risk of death, and the calibration, i.e., the capacity of the model to predict time-to-event, are classical notions for assessing the predictive accuracy, but require specific definitions in the context of dynamic prediction. We use the definition of time-dependent AUC corresponding to a cumulative sensitivity and a dynamic specificity [19, 20] and the definition of time-dependent Brier score (BS) presented in Schoop et al. [21]. At landmark time *s* and for a prediction horizon *t*, these metrics are defined as follows:

$${} {{\begin{aligned} AUC\!\left(s,t\right)=\mathbf{P}\!\left(\pi_{i}\left(s\,+\,t\mathrel{\left|\right.\kern-\nulldelimiterspace}s\right)>\pi_{j}\left(s+t\mathrel{\left|\right.\kern-\nulldelimiterspace}s\right)\!|\ s<X_{i}<s+t,X_{j}>s+t\right) \end{aligned}}} $$

$$BS\left(s,t\right)={\mathbf E}\left[{\left({\mathbf{1}}_{\left\{s<X<s+t\right\}}-\pi(s+t|s)\right)}^{2}|X>s\right] $$

In the context of dynamic prediction, AUC measures the capacity of the model prediction *π*_*i*_(*s*+*t*|*s*) to distinguish between patients of low and high risk of death in the horizon time *t*, while BS measures the average discrepancy between vital status and the prediction in a patient. For AUC, the larger the better, whereas for BS, the smaller the better. With these definitions, a dummy model such that *π*_*i*_(*s*+*t*|*s*)=0.5 for all *i*, *s* and *t* will lead to AUC=0.5 and BS=0.25. Note that AUC does not depend on the number of events while BS does. Therefore, the BS obtained with different landmark times *s* cannot be directly compared [15]. In order to compare BS values over time, one can use a scaled Brier score (sBS) defined in [16, 22]:

$$sBS\left(s,t\right)=\frac{{BS}_{KM}\left(s,t\right)-BS(s,t)}{{BS}_{KM}\left(s,t\right)} $$

where *B* *S*_*KM*_(*s*,*t*) is the Brier score obtained with Kaplan-Meier estimates of the survival function in the training dataset. Thus *s* *B* *S*(*s*,*t*) measures the improvement in model prediction over a prediction that could be done using only the information from the training dataset (the larger the better).

To estimate these metrics, we use weighted estimators to account for right censoring using Inverse Probability of Censoring Weights (IPCW) [23, 24]. Thus the IPCW estimators are:

$${{} \begin{aligned} &\widehat{AUC}\left(s,t\right)=\\ &\quad\frac{\sum^{N}_{i=1} {\!\sum^{N}_{j\,=\,1}{{\mathbf{1}}_{\!\left\{{\hat{\pi}}_{i} \left(s+t\mathrel{\left|\right.\kern-\nulldelimiterspace}s\right)>{\hat{\pi}}_{j}\left(s+t\mathrel{\left|\right.\kern-\nulldelimiterspace}s\right)\right\}\!}{\tilde{D}}_{i}(s,t)(1\,-\,{\tilde{D}}_{j}\left(s,t\right)){\hat{W}}_{i}(s,t){\hat{W}}_{j}(s,t)}}}{\sum^{N}_{i=1}{\sum^{N}_{j=1}{{\tilde{D}}_{i}(s,t)(1-{\tilde{D}}_{j}\left(s,t\right)){\hat{W}}_{i}(s,t){\hat{W}}_{j}(s,t)}}} \end{aligned}} $$

and:

$${} \widehat{BS}\left(s,t\right)\,=\,\!\frac{1}{\sum^{N}_{i=1}{{\mathbf{1}}_{\{T_{i}>s\}}}}\sum\limits^{N}_{i=1}{{\hat{W}}_{i}(s,t){({\tilde{D}}_{i}\left(s,t\right)\,-\,{\hat{\pi}}_{i}\left(s\,+\,t\mathrel{\left|\vphantom{s+t s}\right.\kern-\nulldelimiterspace}s\right))}^{2}} $$

where ${\tilde {D}}_{i}\left (s,t\right)={\mathbf {1}}_{\{s<T_{i}\le s+t\}}$ and the weights ${\hat {W}}_{i}\left (s,t\right)=\frac {{\mathbf {1}}_{\{T_{i}>s+t\}}}{\hat {G}(s+t|s)}+\frac {{\tilde {D}}_{i}\left (s,t\right)\delta _{i}}{\hat {G}(T_{i}|s)}$ take into account censor, with $\hat {G}(u)$ the Kaplan-Meier estimator of survival function of the censoring time at time *u*, i.e., **P**(*C*>*u*) and ∀*u*>*s*, $\hat {G}\left (u\mathrel {\left |\vphantom {u s}\right.\kern -\nulldelimiterspace }s\right)={\hat {G}(u)}/{\hat {G}(s)}$ and ${\hat {\pi }}_{i}\left (s+t\mathrel {\left |\vphantom {s+t s}\right.\kern -\nulldelimiterspace }s\right)$ is defined in the formula (5). Thus, once ${\hat {\pi }}_{i}\left (s+t\mathrel {\left |\vphantom {s+t s}\right.\kern -\nulldelimiterspace }s\right)$ has been obtained in a dataset of *N* ^′^ new patients, as AUC and BS are model free, they can be calculated using packages developed in the context of linear models, and here we use the package timeROC of R [25].

The scaled Brier score is obtained using the estimated Brier score: $\widehat {sBS}(s,t)=\frac {\widehat {BS}_{KM}(s,t)-{\widehat {BS}(s,t)}}{{\widehat {BS}}_{KM}(s,t)}$ where ${\widehat {BS}}_{KM}(s,t)$ is the Brier Score obtained using the Kaplan-Meier estimate at *s*+*t* in the training dataset.

### Application to risk of death in patients with metastatic prostate cancer

Illustration focuses on metastatic Castration-Resistant Prostate Cancer (mCRPC) for which PSA is frequently measured and survival is the primary endpoint. First we develop a reference nonlinear joint model on a training dataset, second we simulate mCRPC patients to evaluate dynamic predictions when the model is known and last we apply this approach to real mCRPC patients from a validation dataset.

#### Building a reference nonlinear joint model

Real data come from mCRPC patients of the control arm of a phase III clinical trial [17] that included 598 men treated with the first-line reference chemotherapy (docetaxel in combination with prednisone). All PSA measurements at baseline (i.e., measured within 8 days before treatment initiation) and after treatment initiation are used. Two patients that have no PSA measurements are not included in the analysis. PSA was measured every 3 weeks during treatment and every 12 weeks after treatment cessation, and the date of death or last visit was obtained for all patients. Data are randomly split into two datasets as described in [26]: a training dataset containing *N*=400 patients to develop the reference nonlinear joint model and to estimate the population parameters *θ*, and a validation dataset containing the *N* ^′^=196 remaining patients to provide individual dynamic predictions and assess the predictability of the model on real data.

In order to describe the kinetics of PSA, we use the biexponential model already described in [9]. In brief, this model assumes that PSA is produced by prostatic cells and that chemotherapy inhibits prostatic cells proliferation with a constant effectiveness *ε* until the time *T*_*esc*_. This leads to the following analytical solution for PSA kinetics:

6 $$ PSA\left(t,\psi\right)=\left\{ \begin{array}{lc} \frac{\delta{PSA}_{0}}{r\left(1-\varepsilon\right)-d+\delta}\ e^{\left(r\left(1-\varepsilon\right)-d\right)t}+\left[{PSA}_{0}-\frac{\delta{PSA}_{0}}{r\left(1-\varepsilon\right)-d+\delta}\right]e^{-\delta t} & if\ t\leq T_{esc} \\ \frac{\delta{PSA}_{0}}{r-d+\delta}\ e^{\left(r-d\right)t-{r\varepsilon T}_{esc}}+ \left[PSA\left(T_{esc,{\psi}}\right)-\frac{\delta{PSA}_{0}e^{(r\left(1-\varepsilon\right)-d)T_{esc}}}{r-d+\delta}\right]e^{-\delta(t-T_{esc})}\ & if\ t>T_{esc} \end{array} \right.  $$

where *P* *S* *A*_0_ (ng.mL ^−1^) is the PSA value at treatment initiation, *δ* (day ^−1^) is the rate of PSA elimination, *r* (day ^−1^) the rate of prostatic cells proliferation in absence of treatment and *d* (day ^−1^) the rate of prostatic cells elimination. *ε* is the constant treatment effect and *T*_*esc*_ the time at which treatment has no longer an effect.

Because only 4 parameters can be identified from Eq. (6), we fix *d* to 0.046 day ^−1^, corresponding to a half-life of tumor cells of 15 days, consistent with an apoptotic index of 5% in metastatic prostate cancer [27]. Moreover we fix *δ* to 0.23 day ^−1^, corresponding to a PSA half-life in blood of about 3 days [28]. Finally PSA kinetics is defined by the vector of parameters *ψ*={*r*, *P* *S* *A*_0_, *ε*, *T*_*esc*_}.

Here in the NLMEM (Eq. (1)), the observed biomarker *y*_*ij*_ corresponds to the *j* ^*th*^ measurement of log(*P* *S* *A*+1) for the patient *i* at time *t*_*ij*_ and *b*(*t*,*ψ*) is log(*P* *S* *A*(*t*,*ψ*)+1), consistent transformation with an additive residual error. Log-normal distributions for *r*, *P* *S* *A*_0_ and *T*_*esc*_ (i.e. *ψ*_*i*_= log(*μ*)+*η*_*i*_) and logit-normal distribution for *ε* (i.e. *ψ*_*i*_=*l* *o* *g* *i* *t*(*μ*)+*η*_*i*_ with $logit(x)=log\left (\frac {x}{1-x}\right)$ for 0<*x*<1) are assumed. The variance-covariance matrix of the random effects is diagonal with parameters: $\Omega = diag\left (\omega ^{2}_{r}, \omega ^{2}_{PSA_{0}}, \omega ^{2}_{\varepsilon }, \omega ^{2}_{T_{esc}}\right)$.

For the survival process, we use a Weibull function for the baseline hazard function $\left (h_{0}(t)=\frac {k}{\lambda }\left (\frac {t}{\lambda }\right)^{k-1}\right)$; further, as no covariate is found statistically significant in univariate selection using a parametric Weibull survival model [26], no covariate is included in the survival model (*γ*=0). Lastly different models of link between PSA and survival are tested:

- No link: *f*(*t*, *ψ*_*i*_)=0
- Current PSA: *f*(*t*, *ψ*_*i*_)= log(*P* *S* *A*(*t*, *ψ*_*i*_)+1)
- Current PSA slope: $f\left (t,\ \psi _{i}\right)=\frac {d\log (PSA\left (t,\ \psi _{i}\right)+1)}{dt}$
- Area under PSA: $f\left (t,\ \psi _{i}\right)=\int _{0}^{t} \log (PSA\left (u,\ \psi _{i}\right)+1)du$

The joint likelihood is maximized using the SAEM algorithm implemented in the software Monolix version 4.3.2. Model selection is based on BIC and the best model is evaluated using residuals for longitudinal (Individual weighted residuals (IWRES)) and survival (Cox-Snell and Martingale residuals) parts and by plotting the mean survival curves compared to the Kaplan-Meier curve in the training and validation datasets [26] (see Additional file 1). This model is called the “reference nonlinear joint model” hereafter and parameters are given in Table 1.

**Table 1:** BIC and parameters estimates (r.s.e (%)) of PSA kinetics and survival in the *N*=400 patients for the 4 joint models

Models	No link	Current PSA	PSA slope	Area under PSA
BIC	14350	14192	14291	14327
*r* (day^−1^)	0.054 (1)	0.054 (1)	0.055 (1)	0.054 (1)
*P* *S* *A*_0_(ng.mL^−1^)	74.6 (8)	73.9 (8)	73.4 (8)	74.9 (8)
*ε*	0.35 (5)	0.34 (5)	0.35 (5)	0.35 (5)
*T*_*esc*_ (day)	138 (4)	138 (4)	142 (4)	136 (4)
*λ* (day)	885 (4)	3800 (9)	1500 (9)	1410 (13)
*k*	1.52 (3)	1.19 (1)	1.33 (9)	1.15 (7)
*β*	-	0.32 (4)	100 (10)	0.00025 (20)
*ω*_*r*_	0.098 (5)	0.098 (4)	0.11 (5)	0.10 (5)
$\omega _{PSA_{0}}$	1.57 (4)	1.57 (4)	1.55 (4)	1.56 (4)
*ω*_*ε*_	1.35 (5)	1.34 (5)	1.22 (5)	1.36 (5)
$\omega _{T_{esc}}$	0.68 (5)	0.64 (5)	0.63 (5)	0.66 (5)
*σ*	0.38 (1)	0.38 (1)	0.38 (1)	0.38 (1)

#### Simulation

PSA and time-to-death are simulated for *N*_*sim*_=200 patients using the vector *θ* estimated with the reference nonlinear joint model. PSA was measured every 3 weeks for 30 months or until the simulated time-to-death, if it occurs before. No other mechanism than death is considered for dropout. Dynamic individual predictions are performed using Stan and R (codes available as Additional file 2) as explained previously in the general framework with *L*=200 the number of Monte Carlo samples. For each landmark *s*∈{0,6,12,18} months and each horizon time *t*>2 months, we calculate the coverage probabilities of the 95% prediction intervals for both PSA and hazard rate, i.e., the proportion of simulated patients for whom the true value of interest (either simulated PSA value or the simulated risk of death) is contained in the corresponding Monte Carlo 95% prediction interval.

Then, using the package timeROC of R [25] we estimate time-dependent AUC, BS and sBS for each landmark time *s* and horizon time *t*, using the Kaplan-Meier estimates computed in the *N*_*sim*_=200 simulated patients themselves to calculate *B* *S*_*KM*_(*s*,*t*).

#### Real data

Individual dynamic predictions are calculated following the same approach than in the simulation in the *N* ^′^=196 mCRPC patients of the validation dataset using the reference nonlinear joint model and *L*=200. Likewise, time-dependent AUC, BS and sBS are estimated using the *N* ^′^=196 real patients. For *B* *S*_*KM*_(*s*,*t*) the Kaplan-Meier estimates in the training dataset are used.

## Results

### Reference nonlinear joint model

All PSA measurements and vital status in the *N*=400 patients from the treatment initiation to the end of follow-up are used to estimate the population parameters of the 4 proposed joint models. Overall 5 710 PSA measurements are used with median [minimum; maximum] number of measurements per patient of 13 [1 ; 55]. Regarding survival, 286 deaths occur (71.5%), leading to a median survival [Kaplan-Meier 95% confidence interval] of 656 days [598 ; 741].

Compared to the different forms of link between PSA and risk of death, the joint model relying on the current PSA is found to have the lowest BIC (Table 1). Thus the link function can be written as follows:

$$f\left(t,\ \psi_{i}\right)=\text{log} (PSA\left(t,\ \psi_{i}\right)+1) $$

All parameters are precisely estimated with relative standard errors (r.s.e) smaller than 9% for both fixed effects and variance components (Table 1), and thus the uncertainty on *θ* is neglected in the following. Details of the model evaluation (individual fits, residuals for longitudinal and survival processes and mean survival curves for both training and validation datasets) are provided in the Additional file 1.

### Simulated data

**Fig. 1:**
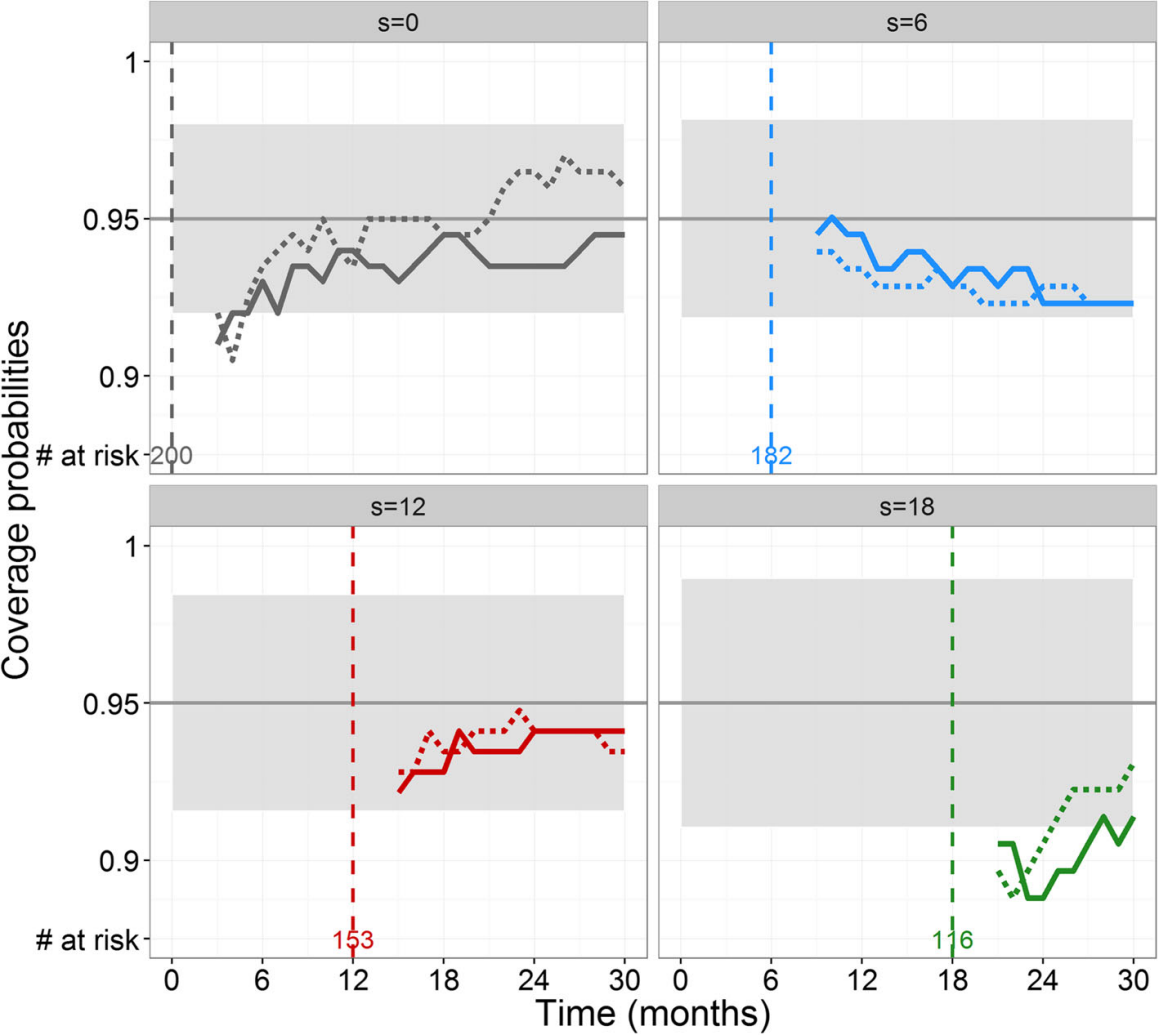
Coverage probabilities of the 95% prediction intervals for PSA values (*dotted lines*) and risk of death (*solid lines*) for 4 values of landmark time *s* (months) and horizon times *t*>2 months in the 200 simulated patients. The 95% prediction intervals of the proportion 95% (*grey areas*) depend on the number of patients at risk which is indicated at bottom at each landmark time *s*

For each simulated patient alive at landmark time *s*, we obtain predicted PSA kinetics and survival probabilities for *t*>2 months with the Monte Carlo 95% prediction intervals. The coverage probabilities of these prediction intervals over the set of horizon time *t* are included in the 95% envelope for both PSA evolution and risk of death for *s*={0, 6, 12} months (Fig. 1). For *s*=18 months, coverage probabilities are smaller than 95%, which suggests that prediction intervals are too narrow.

**Fig. 2:**
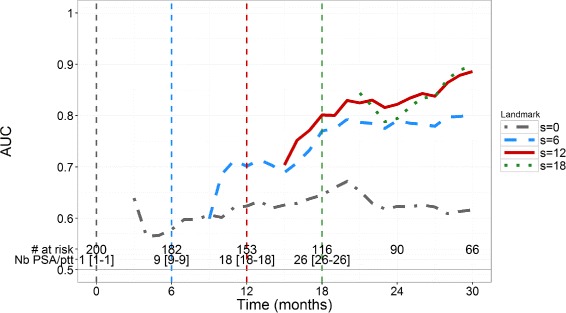
Time-dependent AUC in the 200 simulated patients for 4 values of landmark time *s* (months) and horizon times *t*>2 months. The number of patients at risk in the simulated dataset is indicated at bottom, as well as the median number [minimum-maximum] of PSA observations per patient at risk

For *s*=0, AUC values are about 0.6 (Fig. 2) indicating that the initial PSA value brings some information, but remains of limited use. In order to achieve *A* *U* *C*≥0.7 we find that *s*≥6 months are needed with a horizon time of at least 6 months. In order to achieve *A* *U* *C*≥0.8, a landmark time of at least a year with a horizon time of 6 months is needed to achieve good individual predictions. For example, with *s*=12 months, we find AUC value of 0.80 and 0.82 for horizon *t*=6 and *t*=12 months respectively; with *s*=18 months, AUC values are equal to 0.79 and 0.90 for *t*=6 and *t*=12 months, respectively. For a given landmark time, AUC increases when *t* increases, which indicates that the model better distinguishes patients of low and high risk of death in the long term.

**Fig. 3:**
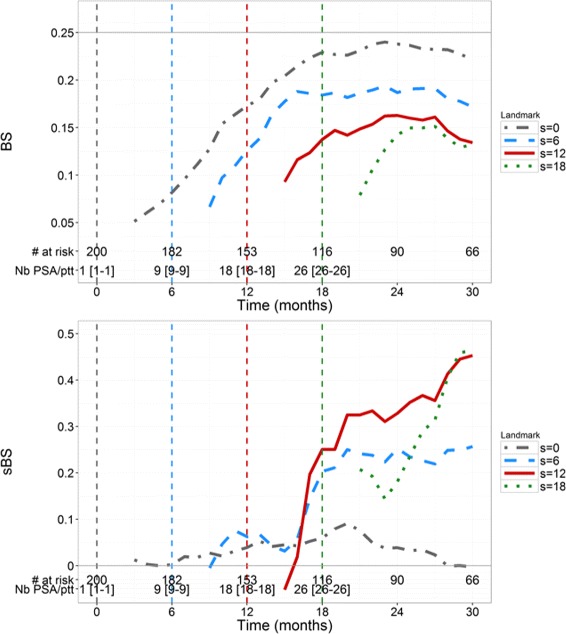
Time-dependent Brier Scores (*top*) and sBS (*bottom*) in the 200 simulated patients for 4 values of landmark time *s* (months) and horizon times *t*>2 months

BS and sBS estimations are provided in Fig. 3. BS for *s*=0 quickly increases (i.e., deteriorates) up to the level of 0.25, which corresponds to a dummy prediction, consistent with the fact that having only baseline PSA provides little information on the time-to-death prediction. For a given landmark time *s*, BS increases when the horizon time *t* increases, consistent with a deterioration of the calibration in long-term prediction. For *s*=12 and *s*=18, BS are smaller than 0.16 and 0.15 respectively, for all horizon times. For *s*=0, sBS remains close to 0 for all horizon times, confirming that baseline value is not sufficient to conduct individual predictions (Fig. 3). For *s*>0, sBS increases (i.e., improves) when *t* increases, even if it never exceeds 0.5. In general, calibration based on individual prediction improves compared to calibration using only Kaplan-Meier estimates, when *s* increases and this becomes particularly true for *s*≥6 and *s*+*t*≥18.

### Real data

In the validation dataset, a total of 2,720 PSA measurements are collected and the median [min ; max] number of measurements per patient is 13 [2 ; 57]. 145 deaths occur (74.0%), with a median survival time [Kaplan-Meier 95% confidence interval] of 598 days [547 ; 732].

**Fig. 4:**
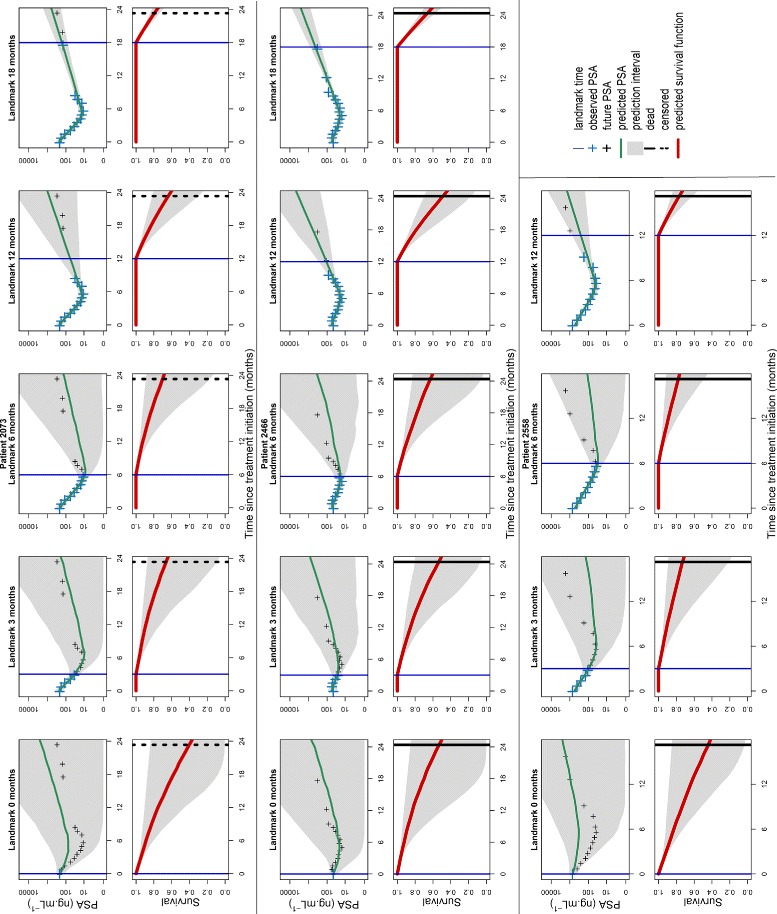
Dynamic individual predictions of PSA evolution and survival for 3 typical mCRPC patients of the validation dataset

Figure 4 illustrates dynamic predictions for 3 typical patients. When the landmark increases, the median prediction of PSA is closer to the future PSA observations with shrinking 95% prediction intervals. In these 3 patients, predictions markedly improve once PSA nadir is attained, due to the fact that all individual parameters can be precisely identified. As a consequence of this uncertainty on PSA future kinetics, the survival function predictions are accompanied by a large 95% prediction interval until the upslope of PSA is clearly observed (landmark times *s*>12 months for patients 2073 and 2466 and landmark times *s*>6 months for patient 2558).

**Fig. 5:**
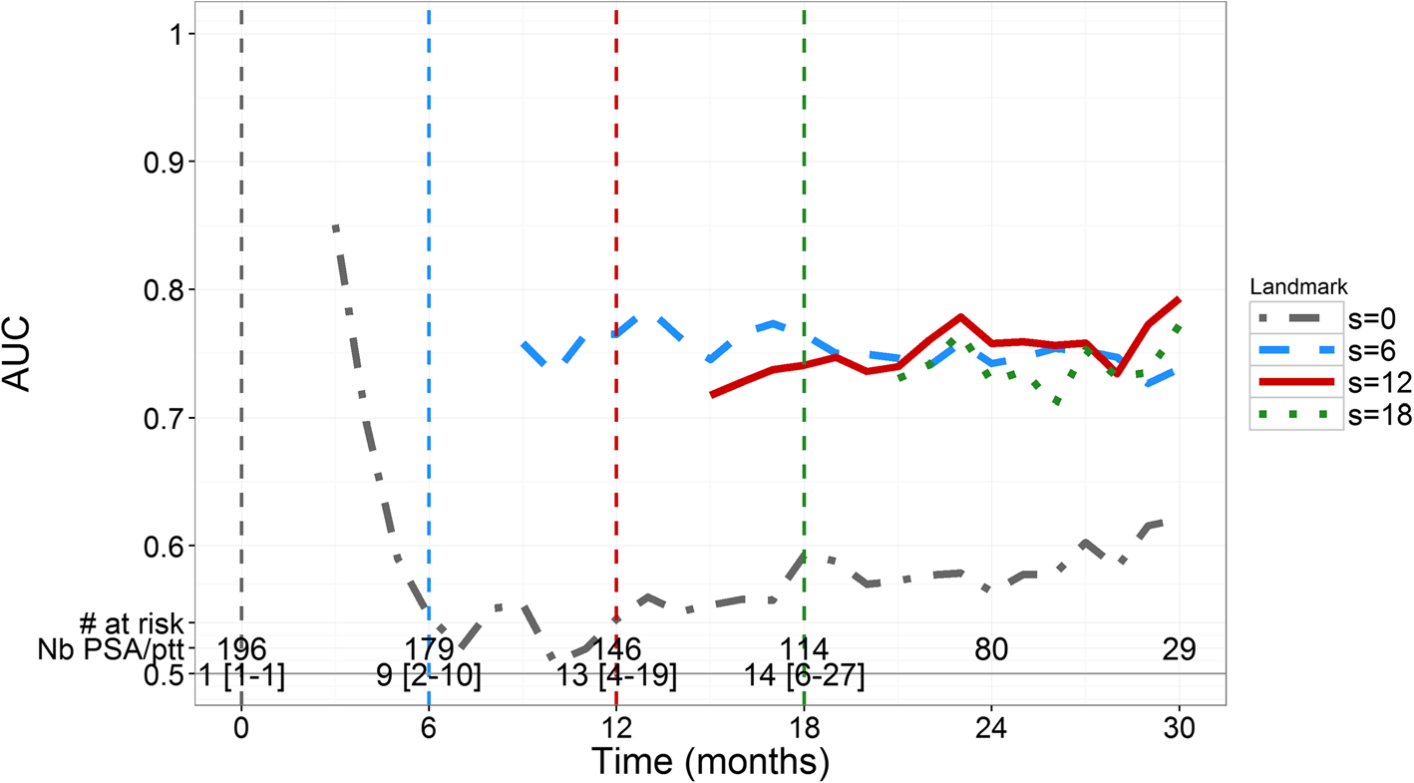
Time-dependent AUC in the *N*’=196 real mCRPC patients of the validation dataset for 4 values of landmark time *s* (months) and horizon times *t*>2 months. The number of patients at risk in the validation dataset is indicated at *bottom*, as well as the median number [minimum-maximum] of PSA observations per patient at risk

Similarly to the simulation study, for *s*=0, AUC values quickly decrease to values lower than 0.6 (Fig. 5). For *s*={6, 12, 18} months, AUC values remain close to 0.75 regardless of the horizon time *t* and do not increase to 0.9 as found in the simulation study. Of note, contrary to the simulation, in real data PSA measurements become less frequent after stopping treatment according to the protocol but patients are still involved in the study and their vital status is collected, which can explain that AUC values remain constant.

**Fig. 6:**
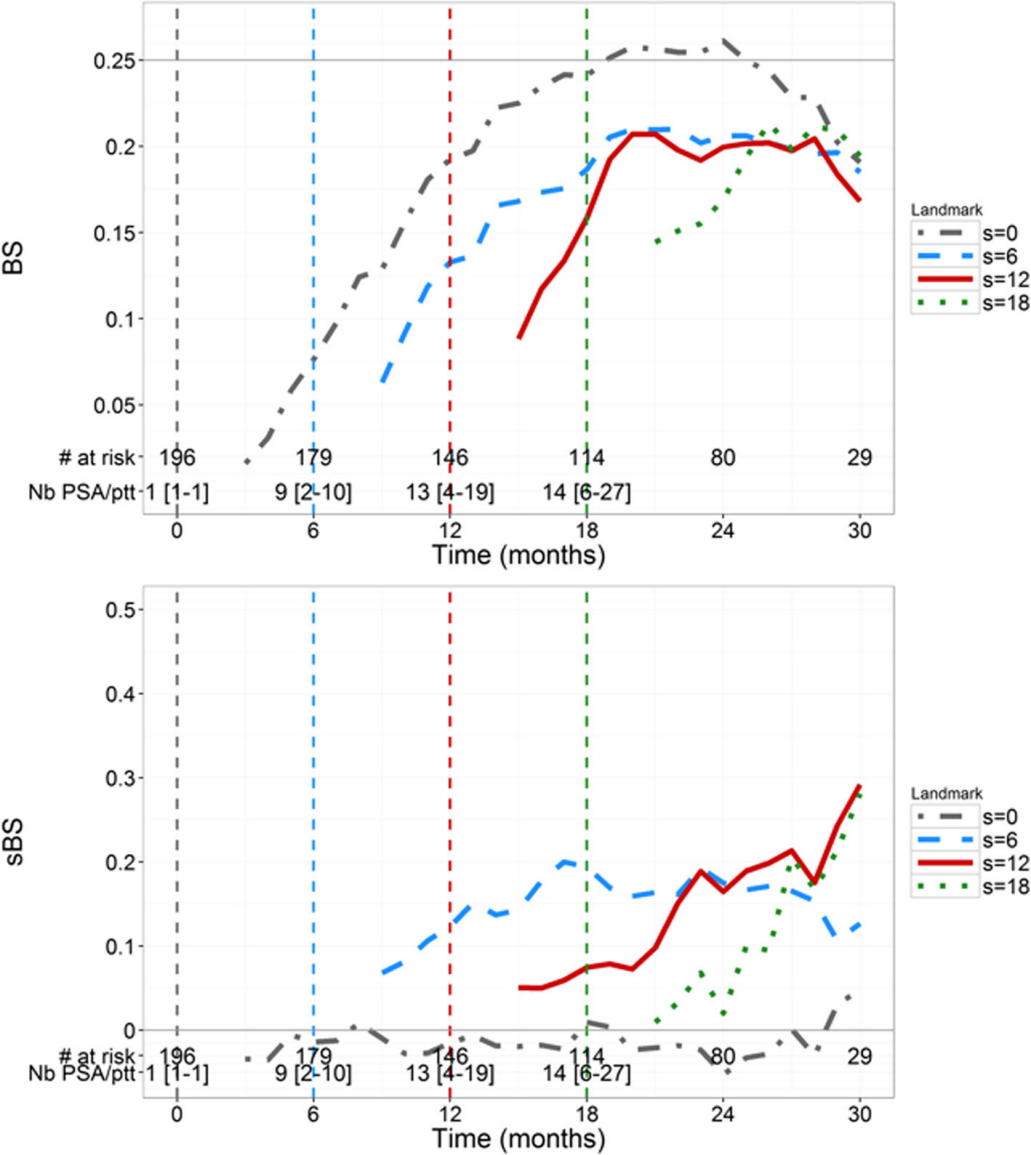
Time-dependent Brier Scores (*top*) and sBS (*bottom*) in the *N*’=196 real mCRPC patients of the validation dataset for 4 values of landmark time *s* (months) and horizon times *t*>2 months

BS and sBS evolutions (Fig. 6) are very similar to those of the simulation. For *s*=0, the rapid increase in BS until 0.25 and using only baseline measurement cannot precisely predict the risk of death at the individual level. For *s*>0, we note that *sBS* is positive meaning that the joint model calibrates better than a Kaplan-Meier estimates. Nevertheless, the *sBS* remain lower to the values found in the simulations (0.29 vs 0.5, respectively), which may be due to lower amount of data over time and to the model limitation itself.

## Discussion

This work is the first one to our knowledge to perform individual dynamic predictions in nonlinear joint models. The following approach is used: i) the priors for the parameters are found by estimating population parameters in a large training dataset using the SAEM algorithm [8, 9], ii) the distribution of the individual parameters is found using the Hamiltonian Monte Carlo (HMC) algorithm and prediction interval for the risk of death is derived accordingly, iii) the predictive performances are assessed using time-dependent discrimination and calibration metrics previously developed in a context of linear model.

Here we use HMC implemented in Stan to characterize the full *a posteriori* distribution of the individual random effects. Of note softwares for nonlinear mixed-effects models (R, SAS or more specifically Monolix or Nonmem in pharmacometrics) can also produce individual “posthoc” parameters, typically the mode (or the mean) of the conditional distribution of the random effects. Yet, in clinical practice, having only the most likely value of the prediction does not account for the uncertainty on the individual parameter estimates. In order to characterize the prediction interval, one frequent approach is to use asymptotic Gaussian approximations [29]. However this may not always be accurate, for instance when the data are limited and additionally it does not take into account the correlations between the parameters. We show by simulation that using HMC implemented in Stan provides good coverage probabilities, except for long follow-up where the prediction interval tended to be overoptimistic (*s*=18 months, see Fig. 1). Whether this is specific to this simulation framework or is a more general pattern will need to be verified. Likewise a formal comparison between HMC and traditional MCMC methods in context of individual dynamic prediction using nonlinear joint model could be of interest. In terms of model prediction assessment, the AUC and BS metrics are model-free and thus can be applied to a nonlinear context using existing packages [25]. Here, while the AUC and the BS improve over the landmark time in the simulation study, they tend to stagnate in the real data. This is likely due to the fact that in the simulation the amount of data increases linearly with the landmark time (since we assume measurements every 3 weeks), while in the real data PSA measurements become less frequent over time in patients after the end of treatment.

Our model framework contains several limitations. First the training and the validation dataset come from the same clinical trial. Second dynamic predictions are performed neglecting the uncertainty on the population parameters *θ*. In our context this approximation is reasonable because the training dataset is large and the relative standard errors are small compared to the between-patient variability (Table 1). However in other contexts this may not be true, for instance in the first steps of adaptive schemes where each new individual is used to update the model prediction. In this case or in external validation, a full Bayesian approach, that can also be done with Stan [30], could be relevant. Further, the biological model, albeit nonlinear, remains very simplistic. For instance effect of covariates like age could be investigated on the longitudinal process. Moreover the model does not account for the mechanisms leading to resistance and then relapse to treatment that we identified previously [26]. Rather we assume that PSA kinetics and risk of death are not modified after treatment cessation and continue at the same pace than before. Moreover PSA kinetics only was assumed to drive the complex process leading to death. These simplifications may explain in part why the model is good at identifying patients at higher risk but does less well at predicting the time-to-death.

## Conclusion

Beside the concrete application shown here, we believe that this approach can be exemplified to develop more biologically relevant models in various medical context. In that respect the recent release of Stan software for stiff ODEs will make possible to use more mechanistic joint models naturally integrating the correlation between several longitudinal processes. Thus, the development of nonlinear models that will accompany the collection of new biomarkers in routine [31] may be an important step towards a better prediction of the risk of death and improve the early identification of patients at greater risk.

## Additional files

Additional file 1 Diagnostic graphs for the reference nonlinear joint model. Individual fits of PSA kinetics and hazard function, residuals for longitudinal and survival parts of the the reference nonlinearjoint model and mean survival curves compared to the Kaplan-Meier curve in the training and validation datasets. (PDF 375 kb)

Additional file 2 Material to provide individual dynamic predictions using Stan. Simulated data, R and Stan codes to draw in the *a posteriori* distribution of the individual parameters, as well as a README document. (RAR 328 kb)
