# Supplementary material for: Nonlinear joint models for individual dynamic prediction of risk of death using Hamiltonian Monte Carlo: application to metastatic prostate cancer
Source: BMC Med Res Methodol. 2017 Jul 17;17:105. doi: 10.1186/s12874-017-0382-9 (PMC5513366; doi:10.1186/s12874-017-0382-9)
Supplement: Supplementary file 1 — Diagnostic graphs for the reference nonlinear joint model. Individual fits of PSA kinetics and hazard function, residuals for longitudinal and survival parts of the the reference nonlinearjoint model and mean survival curves compared to the Kaplan-Meier curve in the training and validation datasets. (PDF 375 kb) [file 12874_2017_382_MOESM1_ESM.pdf]

Additionnal file 1: Diagnostic graphs for the reference nonlinear joint model

Nonlinear joint models for individual dynamic prediction of risk of death using Hamiltonian Monte Carlo: Application to metastatic prostate cancer

Solène Desmée, France Mentré, Christine Veyrat-Follet, Bernard Sébastien, Jérémie Guedj

Once the reference joint model was selected according to the BIC, we carried out a model evaluation which relied on the analysis of the model individual fits and residuals in the training dataset.

First the individual fits were plotted for 9 typical patients, the same than in [1] (see Figure 1). We observed that various PSA kinetics could be well captured by the model.
The residuals for the longitudinal part were assessed using the Individual Weighted Residuals, noted IWRES, defined by:

$$IWRES_{ij} = \frac{y_{ij} - \log(PSA(t_{ij}, \hat{\psi}_i) + 1)}{\hat{\sigma}}$$

where $\hat{\sigma}$ is the estimated standard deviation of the residual error and $\hat{\psi}_i$ is the vector of the estimated individual parameters, i.e., the Empirical Bayes Estimates (EBEs) defined as the mode of the conditional distribution $p(\psi_i|y_i; \hat{\theta})$ with $\hat{\theta}$ the estimation of the population parameters θ. IWRES were plotted versus time and versus predicted PSA values (see Figure 2). This analysis did not suggest any model misspecification in the fitting accross time or PSA values.

The evaluation of the survival part relied on the Cox-Snell and Martingale residuals, noted r_i^{CS} and r_i^M, respectively, defined for $i = 1, ..., N$ by:

$$r_i^{CS} = \int_0^{T_i} h_i(s|PSA(T_i, \hat{\psi}_i))ds$$

$$r_i^M = \delta_i - r_i^{CS}$$

The Kaplan-Meier curve for the r^{CS} was compared to the theoretical survival curve which is the unit exponential distribution $\exp(-t)$, while the Martingale residuals were displayed versus the predicted PSA values (see Figure 2). The Kaplan Meier curve of the Cox-Snell residuals was close to the theoretical survival curve. However there was a slight overestimation of the survival for extrem values of the residuals, indicating a possible underestimation of the risk of death in the patients with a very small or a very high risk of death. Martingale residuals were

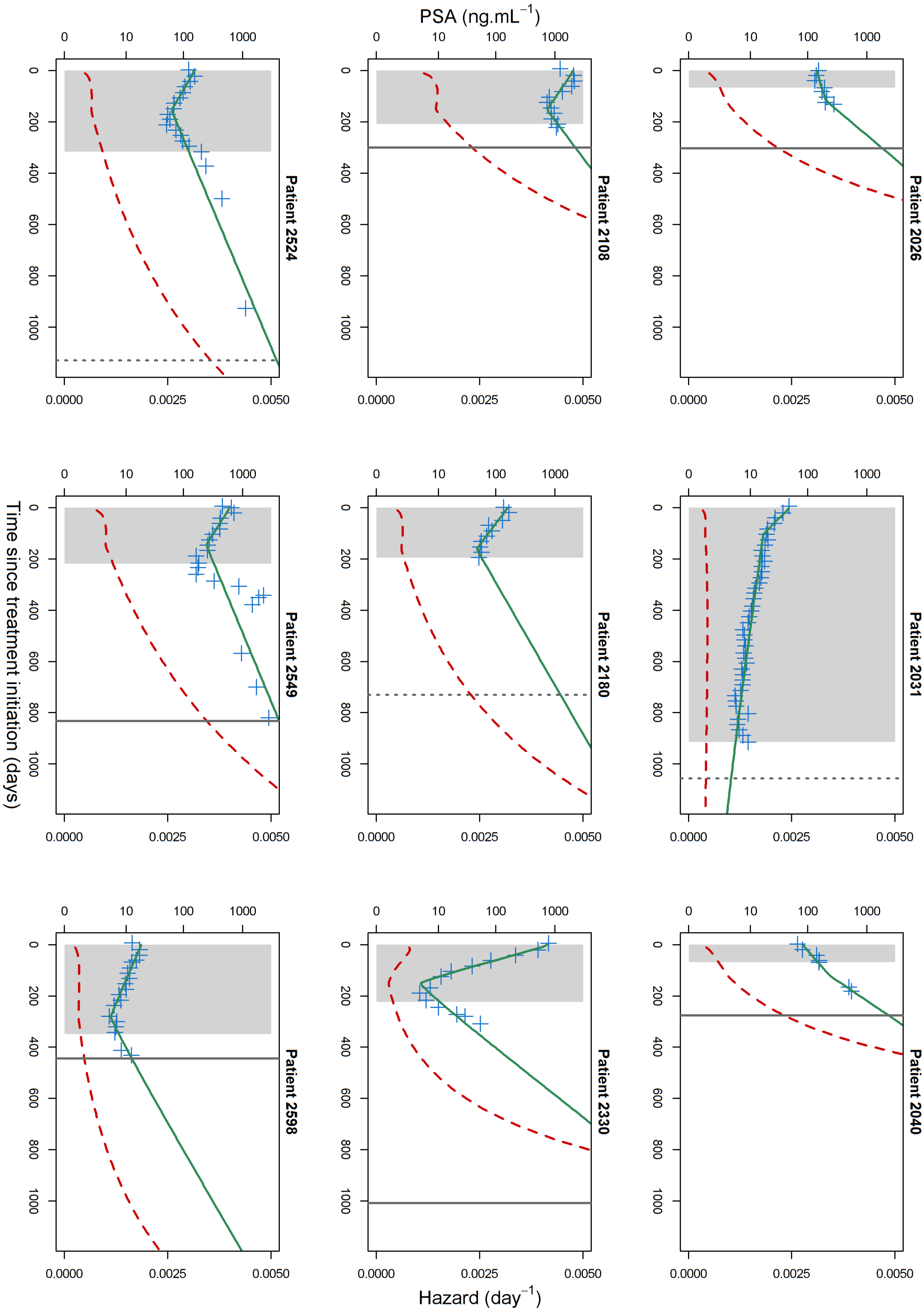

Figure 1: Individual fits of PSA kinetics and hazard function in patients from training dataset with various PSA profiles. Crosses denote PSA measurements and solid lines are the model prediction using the reference joint model. The vertical lines indicate the vital status (solid=death, dotted=censor) at the last recorded time and dashed curves are the individual hazard functions predicted by the reference joint model. The gray area indicates the treatment period.

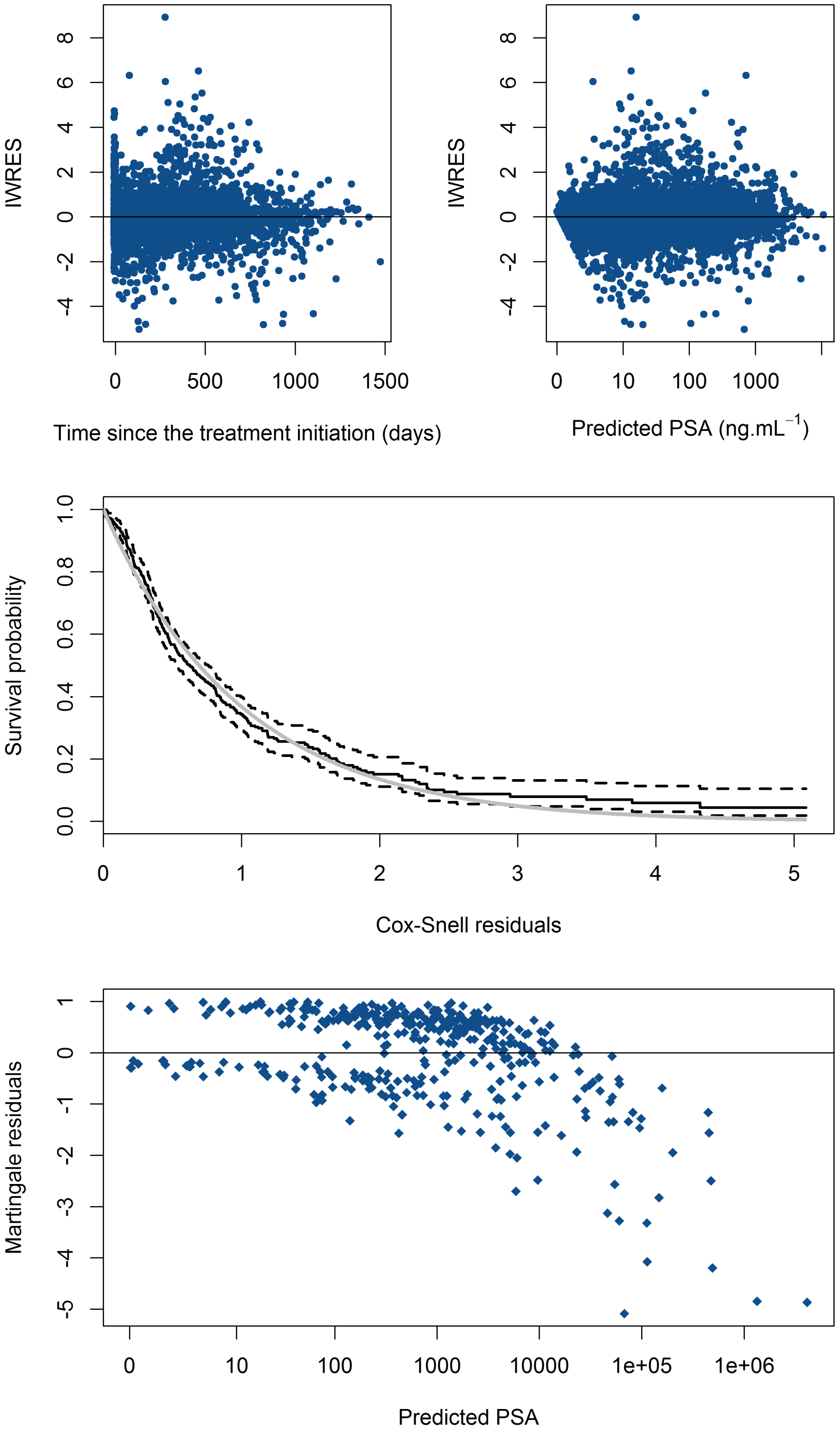

Figure 2: Residuals for the longitudinal and survival parts for the 400 patients included in the training dataset: *Top*: Individual weighted residuals (IWRES) of the longitudinal PSA model versus time (*left*) and versus predicted PSA values (*right*). *Middle*: Kaplan-Meier estimates of the Cox-Snell residuals (solid black line) and its confidence interval (dashed black lines) and the survival function of the unit exponential distribution (solid gray line). *Bottom*: Martingale residuals versus predicted PSA values.

satisfactory according to PSA values for reasonnable values (smaller than 10000 ng.mL^{-1}) but tended to underestimate the survival in patients with large PSA measurements.

In order to evaluate the overall prediction for the survival, the mean survival curve, defined by:

$$S(t) = \frac{1}{N} \sum_{i=1}^{N} S_i(t|PSA(t, \hat{\psi}_i); \hat{\theta})$$

used only the estimated population parameters $\hat{\theta}$ and the longitudinal measurements to estimate the individual parameters (here the EBEs) and to predict survival probability (observed time-to-death are not used). It was compared to the Kaplan-Meier curve for both training and validation datasets (see Figure 3). The mean survival curve was close to the Kaplan-Meier curve for the 2 datasets.

Nevertheless, we can note that this model is less flexible and lead to slightly poorer results than the joint model developed in [1] which relied on a mechanistic system of ordinary differential equations to describe PSA kinetics.

References

[1] Solène Desmée, France Mentré, Christine Veyrat-Follet, Bernard Sébastien, and Jérémie Guedj. Using the SAEM algorithm for mechanistic joint models characterizing the relationship between nonlinear PSA kinetics and survival in prostate cancer patients. *Biometrics*, 2016.

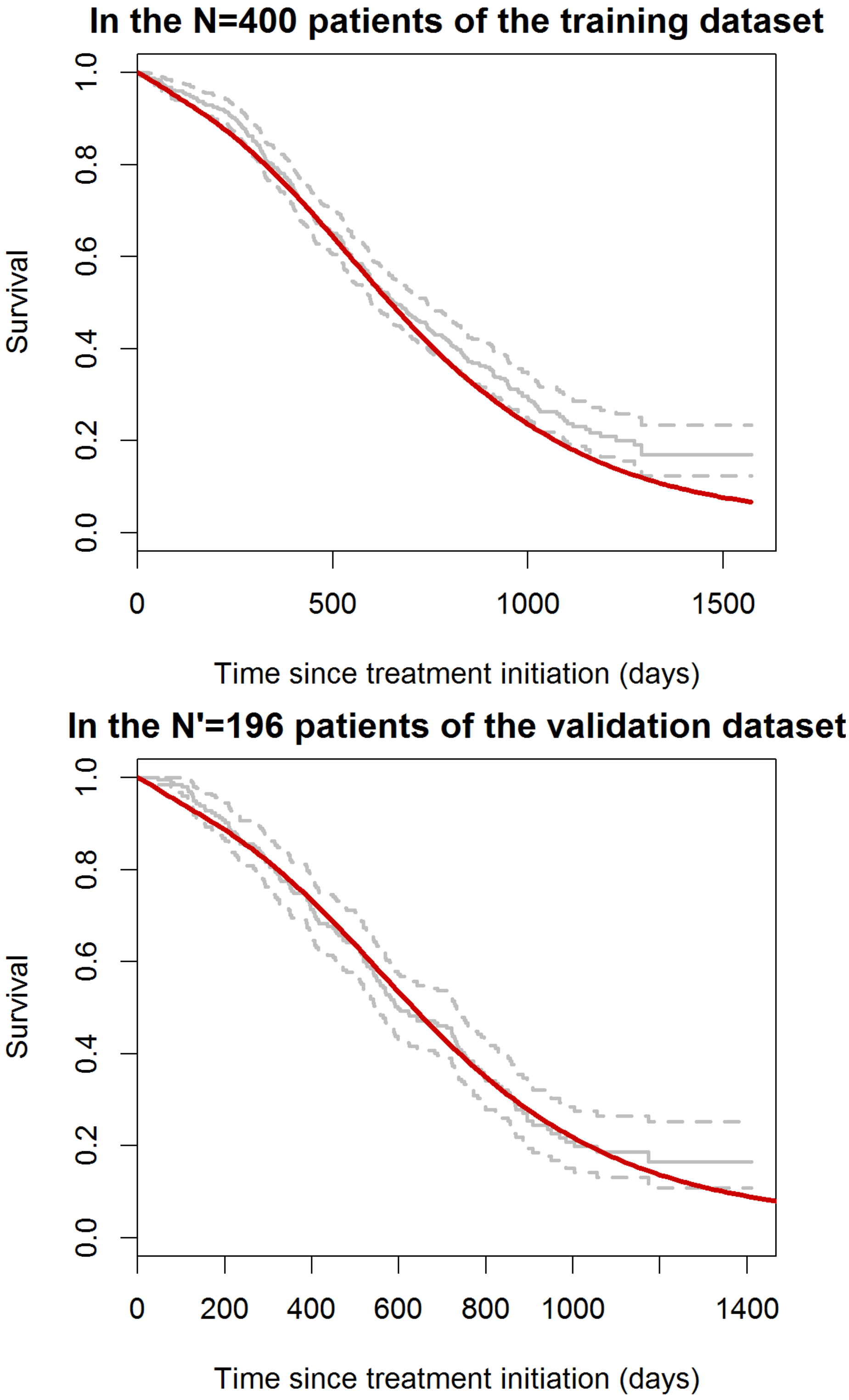

Figure 3: Estimated Kaplan-Meier survival curve (gray solid line) and its confidence interval (gray dashed lines) and mean survival curve (solid line). *Top*: for the 400 patients of the training dataset. *Bottom*: for the 196 patients of the validation dataset
